# Viewpoints of Healthcare Professionals on Care Delivery Within the Frames of Old-Age Mental Telehealth Services Operating in Low-Resource Settings

**DOI:** 10.3390/brainsci15070698

**Published:** 2025-06-28

**Authors:** Eleni Konidari, Emily Adrion, Evaggelia Kontogianni, Maria Alexaki, Eleutheria Aggeletaki, Maria Gkampra, Maria Delatola, Antonis Delatolas, Apostolos Efkarpidis, Gregorios Alokrios, Iοannis Laliotis, Vassiliki Naziri, Anna Petrou, Kalliopi Savvopoulou, Vasileios Stamos, Spiridoula Sideri, Paraskevi Soukouli, Maria Passa, Costas Tsibanis, Theofanis Vorvolakos, Antonios Politis, Panagiotis Alexopoulos

**Affiliations:** 1 Mental Health Services, School of Health Sciences, University of Patras, Rion, 26504 Patras, Greece; elenikonidarimd@gmail.com; 2 Global Health Policy Unit, School of Social and Political Science, University of Edinburgh, Chrystal Macmillan Building, 15a George Square, Edinburgh EH8 9LD, UK; emily.adrion@ed.ac.uk; 3 Global Brain Health Institute, School of Medicine, Trinity College Dublin, The University of Dublin, Lloyd Building Trinity College Dublin, D02 X9W9 Dublin, Ireland; 4 1st Department of Psychiatry, Eginition Hospital, National and Kapodistrian University of Athens, Vasilissis Sophias 72, 11528 Athens, Greece; kontogianni.ev@gmail.com (E.K.); mar.passa@yahoo.com (M.P.); apolitis@med.uoa.gr (A.P.); 5 Primary Healthcare Center of Andros, Chora, 84500 Andros, Greece; alexaki2013@gmail.com; 6 Nursing Services Department, General Hospital of Syros “Vardakeio and Proio”, Geor. Papandreou 2, 84100 Ermoupolis, Greece; eaggeletaki@vardakeio.gov.gr (E.A.); apostolosefkarpidis@yahoo.gr (A.E.); apetrou66@gmail.com (A.P.); 7 Primary Healthcare Center of Xanthi, Andrea Dimitriou 1, 67133 Xanthi, Greece; drmgampra@gmail.com; 8 Primary Healthcare Center of Tinos, Mark. Krikeli 18, 84200 Tinos, Greece; maria.delatola@hotmail.com (M.D.); antonis_kas@hotmail.com (A.D.); 9 Primary Heathcare Center of Chalandritsa, Chalandritsa, 25008 Achaea, Greece; grigorisalokrios@yahoo.gr; 10 Department of Economics, School of Economics and Business, Rion University Campus, 26504 Patras, Greece; ioannis.laliotis@upatras.gr; 11 Primary Healthcare Center of Soufli, Soufli, 68400 Evros, Greece; vnaziri@gmail.com; 12 Primary Healthcare Center of Erymanthia, Erymanthia, 25015 Achaea, Greece; s.poupoulaki@hotmail.com (K.S.); vasileios.stamos@gmail.com (V.S.); 13 Primary Healthcare Center of Katouna, Κatouna, 30004 Aetolia-Akarnania, Greece; siderispiridoula@gmail.com; 14 Primary Healthcare Center of Loutraki, 203000 Corinth, Greece; soukouli3@yahoo.gr; 15 Department of Informatics and Telecommunications, National and Kapodistrian University of Athens, Panepistimiopolis, Ilissia, 15784 Athens, Greece; ktsibanis@uoa.gr; 16 Department of Psychiatry, University General Hospital of Alexandroupolis, School of Health Sciences, Democritus University of Thrace, University Campus, Dragana, 68100 Alexandroupolis, Greece; tvorvola@med.duth.gr; 17 Division of Geriatric Psychiatry and Neuropsychiatry, Department of Psychiatry, Johns Hopkins Medical School, 600 N. Wolfe Street Meyer Building, Baltimore, MD 21287, USA; 18 Department of Psychiatry and Psychotherapy, Klinikum Rechts der Isar, Technical University of Munich, Ismaninger Str. 22, 81675 Munich, Germany; 19 Patras Dementia Day Care Center, Fanariou St. 25, 26226 Patras, Greece

**Keywords:** telemedicine, psychogeriatric care, remote healthcare, healthcare professionals, viewpoints, integrated care model

## Abstract

**Background/Objectives:** The INTegRated InterveNtion of pSychogerIatric Care (INTRINSIC) network introduces an innovative model of psychogeriatric care, combining tertiary mental healthcare with primary care for older adults in low-resource settings in Greece via telemedicine. This study explores viewpoints of healthcare professionals on care delivery within the frames of old-age mental telehealth services in low-resource settings. **Methods:** All healthcare professionals, including 13 medical and 11 non-medical professionals from diverse healthcare units in urban, rural, and insular areas, participated in a semi-structured survey. Thematic analysis identified key insights. **Results:** Most participants (*N* = 19) highlighted the high usability of the INTRINSIC services and their high satisfaction for being members of the network (*N* = 17) was attributed to the collaborative delivery of integrated, specialized healthcare services in primary healthcare (*N* = 17). Further identified advantages of the services included the positive impact on timely care delivery (*N* = 6), cost effectiveness, and alleviation of hospital strain. Healthcare professionals valued the holistic approach of the INTRINSIC services to psychogeriatric care (*N* = 8) and their role in the improvement of it in communities in low-resource settings (*N* = 13). However, challenges were also reported, including the low openness and reluctance of service users (*N* = 7), difficulties in using the INTRINSIC digital platform (*N* = 5), and increased workload (*N* = 5). **Conclusions:** Despite these issues, the INTRINSIC services embody an innovative telehealth model for delivering high-quality, tertiary, mental, and cognitive healthcare services to older adults in underserved areas.

## 1. Introduction

The global population is aging rapidly, with older adults projected to exceed 1.6 billion by 2050 [1]. This demographic shift is most pronounced in low- and middle-income countries, where healthcare infrastructure is often under-resourced [2,3]. Dementia affects over 55 million people worldwide [4] while the prevalence of depressive symptoms varies from 7% to 81% in older adults [5,6,7,8]. The burden of dementia and depression underscores the need for innovative interventions, especially in regions with limited access to healthcare services where over 60% of individuals with dementia and geriatric depression reside [2,3,9]. Of note, we recently detected depressive symptoms and/or cognitive deficits in 58.3% and 76.8% of low-income, homebound, middle-aged, and older adults who reside in rural and semi-urban areas of south-western Greece, a part of the country which includes two regions having a share of people at risk of poverty above 25%, while the prevalence of depressive symptoms and cognitive decline in older individuals in Greece was found to be 19.5% and 18.1%, respectively [8,10,11,12]. These findings are in line with the existing research, which has consistently highlighted the challenges in recognizing and treating depression and cognitive decline among older individuals in low-resource settings. Primary healthcare systems are central to address the mental and cognitive healthcare needs of older adults in low-resource settings, as they provide frontline care and could play a critical role in the timely detection and management of neuropsychiatric disorders [13,14].

The integration of telemedicine into psychogeriatric care has been identified as a promising approach to improve the mental and cognitive healthcare of older people. Telehealth programs have demonstrated positive outcomes in clinical practice, including improved patient health, cost-effectiveness, and satisfaction among both patients and healthcare providers [15,16]. Particularly telepsychogeriatrics, within the primary healthcare setting, has the potential to significantly reduce the strain on secondary and tertiary units and simultaneously lower overall healthcare costs [2,17], since it safeguards access to old-age psychiatry evaluation without traveling for medical consultations, minimizes waiting times, and alleviates the burden of transportation for individuals with multiple comorbidities [17].

The INTegRated InterveNtion of pSychogerIatric Care (INTRINSIC) services are telehealth-based mental and cognitive health services for older adults provided at primary healthcare centers in low-resource settings in Greece [18]. They were founded in 2022 according to a governmental plan for improving mental healthcare for people living in remote areas [19]. Consisting of cooperating primary healthcare centers and old age psychiatry units of university hospitals, the INTRINSIC services aim at timely detection, monitoring, and management of age-related brain diseases among older adults in such areas. The INTRINSIC services rely on six key pillars [19], i.e., (i) a digital platform, enabling synchronous and asynchronous communication between the involved healthcare professionals, as well as the assessment and counseling of the INTRINSIC service users while they visit their primary healthcare therapists, (ii) a comprehensive surveillance system for cognitive, behavioral, and mental health risk factors in older adults, (iii) auditory and vision assessments, performed by primary healthcare professionals trained by sensory therapists, (iv) pharmacological and psychosocial support, (v) a pragmatic psychotherapeutic intervention based on modified problem adaptation therapy (M-PATH) [20], and (vi) community involvement in designing and adjusting the services to local community needs. Prior to their involvement in the INTRINSIC services, healthcare professionals complete a ten-hour, pragmatic, structured online interactive training course focusing on issues related to the diagnosis and post-diagnostic care of mental and/or neurocognitive disorders in aging. The course is organized by the staff of involved old-age psychiatry units. So far, the network consists of eleven primary healthcare centers, each of which is interconnected to one of the three university hospital-based old-age psychiatry units involved in the services. Psychogeriatric care is provided to more than 1100 older adults residing in the catchment areas of the participating primary healthcare centers. Interestingly, new diagnoses of mental and/or neurocognitive disorders were established in approximately 50% of service users [18,19], while use of the services was prematurely terminated by less than 5% of users.

Healthcare professionals involved in the implementation of tele-medicine emphasize its feasibility, flexibility, cost effectiveness, service-user friendliness, and improved accessibility [21,22,23], making it a valuable tool in healthcare delivery. The aim of this study is to investigate the viewpoints of healthcare professionals involved in the INTRINSIC services on the INTRINSIC service’s characteristics and contribution to the management of cognitive and mental healthcare issues of older people residing in low-resource settings in Greece based on their experiences.

## 2. Materials and Methods

### 2.1. Participants and Data Collection

All healthcare professionals involved in the INTRINSIC services were asked to participate in the survey. They were invited via email, which included information about the study and an electronic questionnaire. Data collection took place between December 2023 and March 2024 and was based on an online questionnaire, ensuring anonymity to promote honest and unbiased responses. Completing the questionnaire took approximately ten minutes. Participation in the study was voluntary and anonymous, ensuring the confidentiality of the responses. The study adhered to the ethical principles for research involving human participants, including informed consent and data protection. All healthcare professionals working for the INTRINSIC services in the field have completed the questionnaire and provided feedback.

### 2.2. Questionnaire Content

No validated questionnaires assessing perceptions around telepsychiatry/telehealth from a provider perspective are available (there are a number available examining the patient perspective [24,25,26,27]). Drawing on questionnaire items employed in existing global surveys investigating healthcare provider perspectives on telehealth [28,29,30,31], a semi-structured questionnaire, consisting of closed-ended questions to quantify healthcare professionals’ perspectives and open-ended questions to capture qualitative insights, was developed. This approach grounded the survey in existing, pre-tested survey measures for our key variables/questions, but also allowed room to tailor these to the specific case of the INTRINSIC service. This was supported by an expert review of the questions for content validation as well as through pilot testing. Demographic data and the characteristics of the involvement of each healthcare professional in the INTRINSIC service were tapped (e.g., healthcare center name, duration (in months) of involvement in the INTRINSIC services). The closed-ended questions were answered based on either 5-point ordinal Likert-type scales or categorical responses to assess the usability of the digital platform, the timeliness of care delivery, the INTRINSIC service’s cost-effectiveness, the effectiveness in addressing mental and cognitive health needs, and overall satisfaction. Open-ended questions allowed participants to elaborate on their responses and provide qualitative feedback on the advantages, challenges, and overall experiences related to their involvement in the INTRINSIC services.

### 2.3. Data Analysis Process

Closed-ended responses are presented as frequencies and offered first insights into healthcare professionals’ viewpoints. Open-ended responses were transcribed and reviewed independently by two members of the research team (EK and PA). They analyzed the questionnaire responses independently, identifying key themes and insights.

A thematic analysis of the open-ended responses was employed to uncover participants’ views. Drawing on Brooks and King’s template style [32], a stepwise approach was adopted:

1. Responses were reviewed to ensure familiarity with the data.
2. Initial codes ranging from a few words to several sentences in length were generated based on recurring words, phrases, or ideas. Coding techniques included manually underlining segments or ideas that were repeated or emphasized by the participants and noting codes.
3. Codes were grouped into overarching themes which were narrower and more focused, such as “advantages of INTRINSIC,” “challenges in implementation,” and “cost effectiveness of the program”.
4. Themes were reviewed and refined to ensure coherence and representativeness of the data through the consensus and refinement reached by the two researchers.

To ensure rigor, Lincoln and Guba’s trustworthiness criteria were applied [33]. Independent coding by two researchers enhanced reliability, and collaborative discussions refined the analysis.

Methodological triangulation combined insights from quantitative and qualitative data to provide comprehensive understanding and enhance the credibility of the findings. Investigator triangulation ensured robustness, with two researchers independently analyzing data and reaching a consensus. Preliminary findings were presented to participants via teleconferencing for feedback, ensuring that the themes and interpretations accurately reflected their experiences and perspectives. Of note was that the main researcher (EK) was uninvolved in the design and implementation of the INTRINSIC services, minimizing conflicts of interest and bias. Regular debriefings and collaborative discussions were conducted to minimize subjective biases and ensure consistency in the data analysis. The study adheres to the Consolidated Criteria for Reporting Qualitative Research (COREQ) [34].

## 3. Results

### 3.1. Sample

The study included all healthcare professionals involved in the INTRINSIC services so far: 13 medical professionals (9 general practitioners and 4 psychiatrists) and 11 non-medical professionals (7 nurses, 3 social workers, and 1 psychologist). They served as employees at healthcare centers in urban (*N* = 10), rural (*N* = 7), and insular (*N* = 7) areas, reflecting the diversity of the reach of the INTRINSIC services. The preponderance of participants (*N* = 17) had been involved in the program for over a year. The characteristics of study participants are presented in Table 1.

### 3.2. Viewpoints of Healthcare Professionals on the INTRINSIC Services

**Figure 1:**
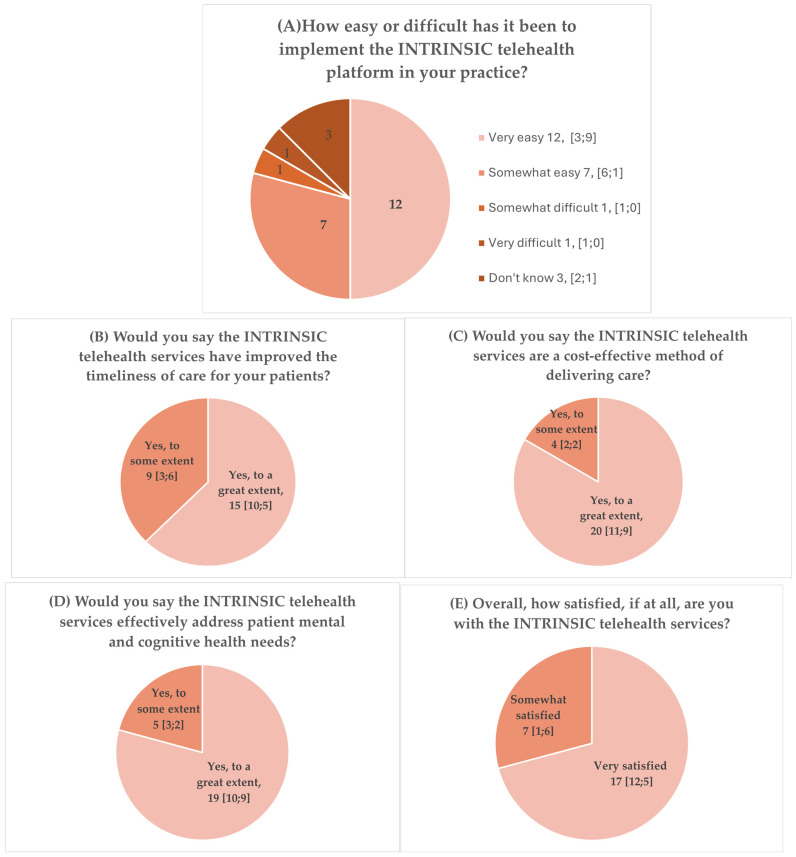
Responses of healthcare professions to closed-ended questions related to their experiences with delivery of psychogeriatric care within the frames of the INTRINSIC services. *N* [medical; non-medical].

The responses of the study participants to the closed-ended items in the questionnaire are summarized in Figure 1.

### 3.3. Usability of the Telehealth Platform

Most of the respondents rated the digital platform as highly or somewhat easy to use and highlighted its intuitive design and seamless integration into existing workflows. One general practitioner remarked, “The platform significantly reduced the time needed to coordinate care, making the process more efficient”. Three healthcare professionals underscored the need for additional training of staff so that its potential could be maximized, and two reported occasional technical difficulties.

### 3.4. Timeliness of Care Delivery

All study participants agreed that the implementation of the INTRINSIC services improved the timeliness of care delivery. Most of them reported significant enhancements in the service delivery, while six underscored the improved access of beneficiaries to healthcare services. Two healthcare professionals from healthcare centers in islandic areas highlighted a critical gap in insular primary healthcare centers, mainly the lack of local general practitioners contributing to the INTRINSIC services primarily due to shortages in medical staff, which was thought to negatively affect the care provided to the INTRINSIC service users in these areas. They suggested that enriching the INTRINSIC teams with medical staff in these areas could further enhance the impact of the services.

### 3.5. Cost-Effectiveness

Twenty healthcare professionals considered the INTRINSIC services cost-effective in delivering cognitive and mental healthcare. The key benefits included reduction in transportation costs for service users (*N* = 7) and decreased overall healthcare expenses (*N* = 3). Three participants noted that the program alleviated hospital strain by enabling remote consultations and the timely initiation of interventions. However, two participants expressed their concerns about the increased workload for healthcare professionals involved in the INTRINSIC services, which may, in the long term, offset some of the cost-saving benefits of the services.

### 3.6. Effectiveness in Addressing Cognitive and Mental Health Needs

Most of the study participants rated the program as highly effective in meeting the cognitive and mental health needs of older people residing in low-resource settings. The benefits included comprehensive cognitive, mental, and behavioral assessments (*N* = 6); opportunities for service-user reassessments (*N* = 2); and the timely initiation of treatment (*N* = 2). The holistic approach of the INTRINSIC services, addressing both medical and psychosocial needs, was particularly valued.

### 3.7. Satisfaction Levels

Satisfaction levels among healthcare professionals were from moderate to high, with 17 participants reporting to be very satisfied and 7 somewhat satisfied. Urban and rural respondents reported higher satisfaction. All study participants described their experience in delivering psychogeriatric care as positive. Thirteen participants attributed their positive experiences to various factors, including the holistic approach of the INTRINSIC services and the provision of specialized, high-quality psychogeriatric care to older individuals living in low-resource settings (*N* = 5), and the psychosocial framing of the needs of service users (*N* = 8), while education and training opportunities in old-age mental healthcare were also appreciated (*N* = 5).

### 3.8. Advantages of the INTRINSIC Services

The focus of the INTRINSIC services on providing specialized, high-quality psychogeriatric care was the most frequently mentioned advantage (*N* = 17), followed by improved accessibility to care for older adults (*N* = 14). Other benefits included the holistic approach (*N* = 9); the timely initiation of interventions (*N* = 6); the reduction in stigma associated with mental disorders (*N* = 3); close, interprofessional collaboration (*N* = 4); and the innovative nature of the services (*N* = 3). One participant highlighted the healthcare transformative impact of the services, stating: “*The integration of specialized psychogeriatric care into primary healthcare delivery has been life-changing for older adults in remote areas*”.

### 3.9. Challenges Encountered During Implementation of the INTRINSIC Services

The most commonly reported challenges were stigma and the reluctance and hesitation of service users (*N* = 7), particularly in rural (*N* = 5) and insular areas, according to the healthcare professionals involved in the services. Technical difficulties (*N* = 5), including the instability of internet connections and low familiarity with the use of the digital platform, were also noted. Interestingly, technical difficulties were mostly mentioned in urban areas (*N* = 4) and not in rural or insular areas. Two study participants mentioned challenges related to cooperation with service users, indicating occasional difficulties in aligning expectations or fostering trust in the diagnostic and therapeutic interventions of the INTRINSIC services. Interestingly, five healthcare professionals reported professional challenges such as fatigue and isolation, especially due to a lack of support from colleagues not involved in the INTRINSIC services. Additional barriers included managing complex cases (*N* = 3), pointing to the need for more in-depth specialized training and access to additional resources (*N* = 1), while one healthcare professional mentioned the reluctance of local private physicians to refer older adults to telehealth services.

Table 2 summarizes the key themes and subthemes identified through qualitative analysis, providing a concise overview of the findings.

### 3.10. Differences Across Professional Roles

The closed-ended questions revealed that non-medical professionals became more easily familiar with the use of the telehealth platform compared with physicians (Figure 1). On the other hand, general practitioners and psychiatrists tended to be more enthusiastic regarding the improvement in the timeliness of care, while both medical and non-medical staff appreciated the cost-effectiveness and effectiveness of the INTRINSIC services in meeting the cognitive and mental health needs of older people residing in low-resource settings. In addition, medical professionals tended to be more satisfied with the services than their non-medical colleagues.

Open-ended response analyses unveiled that the timely diagnosis of mental and cognitive health issues and initiation of the necessary treatment measures, the availability of and accessibility to specialized healthcare services, the holistic approach of the services, the perceived usefulness, and the cost-effectiveness, including reductions in transportation needs, healthcare costs, and hospital strain, were widely acknowledged across all professional groups (Table 3). Of note was that improvements in the evaluation of neuropsychiatric symptoms were specifically mentioned by three general practitioners.

Technical difficulties and uncertainties in the continuity of care and management of complex cases were reported by both medical and non-medical professionals, while the need for greater familiarization with the telehealth digital platform was mentioned by two psychiatrists and one social worker. Medical professionals also highlighted fatigue as an emotional challenge they encountered in their roles. One general practitioner and one social worker considered the program an added burden for healthcare staff, whereas two nurses highlighted the need for close collaboration with medical professionals within the primary healthcare setting. Moreover, three nurses noted limitations in meeting patient needs. Concerns about stigma and hesitance among beneficiaries were mainly raised by five general practitioners but were also mentioned by a nurse and a social worker.

## 4. Discussion

The INTRINSIC services stand out for their novel design that relies on the central role of primary healthcare professionals. They play a central role as mediators between service beneficiaries and old-age psychiatry experts via telemedicine, while they enrich INTRINSIC with their expertise in the field of physical illness prevention and care [18,19], since both mental phenomena and physical aspects crucially affect brain health promotion and maintenance [35,36]. The INTRINSIC design bypasses several barriers commonly encountered in telehealth programs, such as the challenges posed by comorbidities, sensory loss [37], and the technical illiteracy of older adults [38], as well as the inability to conduct physical examinations via telemedicine [39]. From training, close collaboration with old-age psychiatrists via telemedicine, and mounting clinical experiences, primary healthcare providers—both medical and non-medical— acquire the necessary expertise so that the timely detection of cognitive and/or mental issues in aging and the timely initiation of the necessary interventions is enabled in primary healthcare centers, as highlighted by the participants of our study [14]. Timely intervention is critical in psychogeriatric care, where delays in diagnosis or treatment can exacerbate symptoms and lead to poorer outcomes [40], especially in low-resource settings [41]. By integrating psychogeriatric expertise into primary care, the INTRINSIC network addresses this gap effectively [18,19]. The INTRINSIC services have contributed to improvements in care delivery for older adults in low-resource settings, as highlighted by the lived experiences of healthcare professionals involved in them. The findings of our study align with prior research on the benefits of telehealth in enhancing access to care and reducing healthcare disparities, particularly in underserved populations, as shown by studies including socially isolated older people [42,43].

One of the key strengths of the INTRINSIC services, underscored by 19 healthcare professionals involved in them, is the usability of their digital platform. Unlike many telehealth services in which older adults are expected to directly interact with digital platforms, INTRINSIC does not depend on such an interaction. This design mitigates the technical challenges often faced by older adults, particularly by those living with cognitive impairment, such as difficulties navigating digital tools or the lack of familiarity with new technology [44]. The platform’s intuitive design and the training provided to healthcare professionals likely contributed to its success. Training efforts helped most primary healthcare providers to seamlessly integrate the platform into their workflows, enabling more efficient care coordination. These findings are consistent with the existing literature, which emphasizes the importance of user-friendly interfaces and adequate training in telehealth adoption [39,45]. Nonetheless, in many cases the INTRINSIC service staff underlined the need for additional training, so that they could become more familiar with the telehealth platform, overcome the difficulties they encountered with its use, and maximize the potential of the services. Changes in the design of the platform and support by computer technicians [46] may further improve its user-friendliness and may be the topic of a future focus group consisting of the INTRINSIC service healthcare professionals and computer scientists involved in creating the platform.

The design of the INTRINSIC services largely enables the overcoming of the dependency of many telehealth models on the support of care partners of service beneficiaries due to technology-related challenges pertaining to lack of access, low technology literacy, and/or discomfort with new technologies [46]. In fact, in many telehealth models designed for people with cognitive decline, care partners play a pivotal role in facilitating the interaction between older adults and healthcare professionals via mobile phones and tablets [42,43]. Relying on care partners can become a barrier, particularly in cases where care partners are unavailable, lack the necessary skills, or are severely burdened by the care of the service beneficiary. INTRINSIC eliminates this dependency by positioning healthcare providers as the primary users of the digital platform, ensuring that older adults have access to necessary mental and cognitive care regardless of care partner support and availability, since they do not need to interact with smartphones, tablets, or computers in order to have access to the INTRINSIC services.

Healthcare professionals who participated in the study appreciated the holistic approach of INTRINSIC to psychogeriatric care. This approach, noted by one-third of study participants, addresses not only the mental and cognitive needs of older adults but also their medical and psychosocial needs [18,19]. Holistic care is essential in psychogeriatrics, since mental, cognitive, and medical health issues are often intertwined with social and environmental factors [47]. This characteristic of the INTRINSIC services, which rely on multiprofessional teams consisting of medical and non-medical primary healthcare professionals and old-age psychiatrists, is particularly noteworthy given the relatively limited availability of holistic intervention programs for older adults and the plethora of determinants, of different natures, of brain health maintenance and promotion [47]. For instance, the management of hearing- and vision loss, the timely detection and treatment of depression, and the management of metabolic and cardiovascular factors, such as hypertension, dyslipidemia, and diabetes, are pivotal in reducing the risk of cognitive decline and maintaining brain health [48].

The perceived cost-effectiveness of the INTRINSIC services was emphasized by all study participants. The key benefits included reduced transportation costs for older people seeking mental and/or cognitive care, diminished strain on secondary and tertiary healthcare settings, lower risk for hospitalization and the wide spectrum of patient harm related to it (e.g., nosocomial infections, delirium, falls, and transitions of care) [49], and the overall reduction in healthcare expenses. These findings align with the existing research on telehealth, which consistently highlight cost savings as a major advantage [50]. Indeed, telehealth facilitates the detection, monitoring, and management of disorders, functional decline, and other key changes in medical status before acute care in the emergency department or hospital, or long-term care in a nursing facility become inevitable [50]. Moreover, the design of the INTRINSIC services reduces logistical barriers, such as limited access to convenient public transport or adequate access to all-weather roads, and likely further enhances the cost-effectiveness of the services [43].

The overall satisfaction with healthcare involved in INTRINSIC was high, with 17 participants reporting being very satisfied. This level of satisfaction is in line with reports on the viewpoints of healthcare workers involved in telemedicine services for people with Parkinson’s disease or mental disorders in the United States or Ireland, respectively, as well as of professionals across different roles in the healthcare system, including physicians, pharmacists, nurses, and allied health professionals from several public or private practice sites in Kuwait [22,51,52]. Close collaboration among healthcare professionals and the opportunities for education and training, provided by INTRINSIC, were also identified as positive factors contributing to the successful implementation of the services [53]. These aspects fostered a sense of professional growth and teamwork among participants.

Despite their strengths, the implementation of the INTRINSIC services was confronted with several challenges. One of the main barriers was the limited digital literacy of some healthcare professionals, which could have hindered their understanding of the INTRINSIC processes [53,54]. Technical difficulties, including issues with equipment usage [55], hinder the ease of telemedicine adoption, and these challenges are commonly reported by healthcare professionals [18,19,36]. This issue highlights the need for ongoing education and support for healthcare professionals so that access to web-based care for people who otherwise would have been excluded from care is facilitated [39,45]. It was previously highlighted that telehealth providers required specific training focused on recognizing and overcoming their own biases about telehealth [46,56]. The development of standardized national curricula across healthcare training programs has been recommended based on the findings of a comprehensive literature review of telehealth education integrated into the curricula of physician, physician assistant, and advanced practiced registered nurse training programs in the United States [56]. Interestingly, healthcare organizations are increasingly investing in implicit bias trainings [33]. A simple mindset shift can make each clinician no more wonder “who,” but rather “how” they can facilitate a telehealth visit for the users of their services, so that the same access to a valuable component of care is safeguarded [46].

Resource constraints in rural and island settings also posed challenges, particularly in terms of staffing shortages [44]. Healthcare staffing crises, particularly shortages of specialized physicians, and inequity [57] in their distribution are common challenges for healthcare services not only in low-resource settings in high-income countries, but also in the Global South [58,59]. In Greece, shortages of healthcare workers are mainly caused by the intricate interplay of economic disparities, inadequate working conditions, and limited career advancement opportunities. Addressing these issues through evidence-based policies, strategic workforce planning, and transnational cooperations in the near future is pivotal for safeguarding equity in healthcare quality across the globe and will be critical for the INTRINSIC services’ scalability and sustainability as well [44,57]. Quite unexpectedly, technical difficulties were mostly mentioned in urban areas and not in rural or insular areas. Technical difficulties related to limited access to internet and/or system unreliability were mentioned as barriers to the implementation of telehealth services in 12 reviews [60]. The less frequently encountered difficulties in rural and insular areas may reflect advances in the internet infrastructure in these regions, since the access to highspeed connection is thought to represent an incentive for retaining inhabitants and to help repopulate remote areas, giving people the possibility to perform their work from home, without commuting to more densely populated areas [61]. Alternatively, it may mirror differences in the number of challenges related to the INTRINSIC implementation between urban and rural/insular areas with healthcare professionals working in the former being confronted with fewer challenges, so that technical difficulties were mentioned more frequently by them.

Furthermore, healthcare professionals highlighted the challenge of overcoming stigma and the reluctance of beneficiaries to accept help, particularly in rural and insular areas, aligning with findings of previous studies, which have shown that people residing in low-resource settings do not seek help and tend to exhibit high levels of non-take-up of mental health services [46,60]. A number of study participants also reported a potentially increased workload and fatigue associated with being involved in the INTRINSIC services, an issue that remains contested in the literature. While some studies emphasize the fatigue and overload experienced by healthcare providers, others suggest a reduction in workload as a result of telehealth services [22,62,63].

Our study sheds light on differences in the viewpoints between medical and non-medical professionals. The higher satisfaction and enthusiasm of medical professionals regarding the added value of the INTRINSIC services may be attributed to the dominating role of physicians in shaping the design of the services and who subsequently encounter the implementation of the services more positively compared to non-medical staff. In addition, the commonly reported medical dominance in multiprofessional teams [64], time pressure in communication between physicians and service beneficiaries, and paternalism/overprotection may limit the space that is provided to service users for expressing concerns and providing feedback [65]. In contrast, non-medical professionals, i.e., nurses, psychologists, and social workers, spend more time with beneficiaries and can serve as translators or the beneficiaries’ advocates [66]. These differences may explain the relatively lower level of enthusiasm among non-medical INTRINSIC staff, as well as the concerns about stigma and hesitance among beneficiaries, which were mainly raised by physicians. The more frequently reported difficulties of medical healthcare workers with the use of the digital platform compared to non-medical staff may stem from a lack of time in becoming familiar with it [67] and/or to the previously reported low confidence of a portion of the healthcare workforce in using information systems and information and communication technology, despite their positive attitudes towards them [68].

While the INTRINISC services seem to reduce systemic barriers to care, further research employing longitudinal designs, investigating both healthcare workers’ and service users’ perspectives, and/or evaluating long-term outcomes is needed, in order to understand the impact of the services, particularly in the light of the relatively limited number of participants of this study and their relatively short experience with the INTRINSIC services, being still in its infancy. Considering the overwhelmingly positive responses, the possibility of a social desirability bias cannot be excluded, since study participants were involved in the implementation of this pioneer program, even though participation in the study was voluntary and anonymous ensuring the confidentiality of responses. Additionally, it would be valuable to examine the views of large numbers of healthcare providers who have been involved with the INTRINSIC program for an extended period. Future studies should also investigate the impact of the INTRINSIC services on user satisfaction and trust, as well as their effectiveness in managing complex cases. Longitudinal research will be particularly valuable in assessing the INTRINSIC services’ sustainability and cost-effectiveness over time.

## 5. Conclusions

The INTRINSIC services embody a scalable and innovative model for integrating psychogeriatric care into primary healthcare through telemedicine. Providing holistic, team-based, and pragmatic mental and cognitive healthcare to older adults fueled the motivation of the INTRINSIC healthcare professionals and facilitated the implementation of this novel healthcare model. The findings reported here suggest that the INTRINSIC model could serve as an exportable and adaptable framework for other low-resource primary healthcare settings, improving access to pragmatic cognitive and mental health services for older individuals.

## Figures and Tables

**Table 1:** Characteristics of the INTRINSIC services’ staff participating in the study.

Participant Characteristics	Number (*N*)	Percentage/Mean
Total number of participants	24	
**Profession**		
Medical professionals	13	54.2%
General practitioners	9	
Psychiatrists	4	
Non-medical professionals	11	45.8%
Nurses	7	
Social workers	3	
Psychologists	1	
**Months working in the program** [*mean,* (*standard deviation*)]		1.23 (0.429)
More than 12 months	17	
Less than 12 months	5	

**Table 2:** Key Themes and Subthemes Identified from Qualitative Data.

Theme	Subthemes	Illustrative Quotes
**Benefits and opportunities**	Holistic approach Timely interventions Satisfaction Specialized, high-quality care Multiprofessional collaboration	“The integration of psychological and social support into care delivery has been transformative.”
**Challenges and barriers**	Stigma Technical difficulties High workload Complex cases Lack of medical professionals in a number of local INTRINSIC networks Fatigue	“Some patients were hesitant due to mistrust of telehealth.” “Face-to-face practice remains essential for complex cases.” “Insular regions lack adequate medical staff, which may limit the potential of the services.”
**Experience in the field**	Positive Close, multiprofessional collaboration Education and training in psychogeriatric care	“Working with a multidisciplinary team has been a key factor in delivering effective care.”

**Table 3:** Responses of healthcare professionals to open-ended questions regarding their experiences with the INTRINSIC telehealth services.

Key Theme	Medical Professionals (*N* = 13)	Non-Medical Professionals (*N* = 11)
Technical difficulties with the use of the telehealth digital platform	4	2
Need for familiarization with the telehealth digital platform	2	1
Improved healthcare accessibility	8	6
Availability of specialized old-age mental and cognitive healthcare services in primary care in low-resource settings	10	7
Services’ holistic approach	4	5
Emotional strain (fatigue, isolation due to lack of support from colleagues not involved in the INTRINSIC services)	4	1
Stigma among beneficiaries	5	2
Overcoming stigma	2	1
Burden-Workload perception	1	1
Need for medical professional involvement in primary care		2

## Data Availability

The datasets analyzed during the current study are available from the corresponding author on reasonable request. The data are restricted from public access due to privacy and ethical restrictions.

## Abbreviations

The following abbreviations are used in this manuscript:

COVID-19	Coronavirus Disease 2019
INTRINSIC	INTegRated InterveNtion of pSychogerIatric Care
M-PATH	Modified problem adaptation therapy
WHO	World Health Organization
